# Primary Production in the Water Column as Major Structuring Element of the Biogeographical Distribution and Function of Archaea in Deep-Sea Sediments of the Central Pacific Ocean

**DOI:** 10.1155/2019/3717239

**Published:** 2019-03-03

**Authors:** Franziska Wemheuer, Avril Jean Elisabeth von Hoyningen-Huene, Marion Pohlner, Julius Degenhardt, Bert Engelen, Rolf Daniel, Bernd Wemheuer

**Affiliations:** ^1^Genomic and Applied Microbiology and Göttingen Genomics Laboratory, Institute of Microbiology and Genetics, University of Göttingen, Göttingen, Germany; ^2^Applied Marine and Estuarine Ecology, Evolution and Ecology Research Centre, School of Biological, Earth and Environmental Sciences, University of New South Wales, Sydney, Australia; ^3^Paleomicrobiology Group, Institute for Chemistry and Biology of the Marine Environment, University of Oldenburg, Oldenburg, Germany; ^4^Centre for Marine Bio-Innovation, School of Biological, Earth and Environmental Sciences, University of New South Wales, Sydney, Australia

## Abstract

Information on environmental conditions shaping archaeal communities thriving at the seafloor of the central Pacific Ocean is limited. The present study was conducted to investigate the diversity, composition, and function of both entire and potentially active archaeal communities within Pacific deep-sea sediments. For this purpose, sediment samples were taken along the 180° meridian of the central Pacific Ocean. Community composition and diversity were assessed by Illumina tag sequencing targeting archaeal 16S rRNA genes and transcripts. Archaeal communities were dominated by Candidatus *Nitrosopumilus* (*Thaumarchaeota*) and other members of the *Nitrosopumilaceae* (*Thaumarchaeota*), but higher relative abundances of the Marine Group II (*Euryarchaeota*) were observed in the active compared to the entire archaeal community. The composition of the entire and the active archaeal communities was strongly linked to primary production (chlorophyll content), explaining more than 40% of the variance. Furthermore, we found a strong correlation of the entire archaeal community composition to latitude and silicic acid content, while the active community was significantly correlated with primary production and ferric oxide content. We predicted functional profiles from 16S rRNA data to assess archaeal community functions. Latitude was significantly correlated with functional profiles of the entire community, whereas those of the active community were significantly correlated with nitrate and chlorophyll content. The results of the present study provide first insights into benthic archaeal communities in the Pacific Ocean and environmental conditions shaping their diversity, distribution, and function. Additionally, they might serve as a template for further studies investigating archaea colonizing deep-sea sediments.

## 1. Introduction

*Archaea* play a key role in global biogeochemical cycles [1–4]. These microorganisms are widely distributed in various environments such as permafrost [5], hot springs [6, 7], hypersaline lakes [8], marine seawaters [9], and freshwater as well as marine (deep-sea) sediments [10–12]. Deep-sea sediments constitute one of the largest ecosystems on earth, covering approximately 65% of its surface. This ecosystem is characterized by extreme conditions including high pressure, cold temperatures, and lack of light. Nonetheless, deep-sea sediments harbor taxonomically and metabolically diverse archaeal communities [11–16]. These communities are generally dominated by archaeal taxa belonging to the phyla *Thaumarchaeota* and *Euryarchaeota* [11, 13, 16–18].

Given the important ecological role of *Archaea*, it is fundamental to decipher key drivers of diversity, distribution, and function of archaeal communities in marine deep-sea sediments. Previous investigations revealed that these communities are affected by a wide range of environmental conditions. These include sediment depth, distance from land, water depth, site, and latitude [12–15, 19]. Other important factors driving archaeal communities in marine sediments are the hydrography of overlying water masses and/or the quantity and composition of organic matter [11, 13, 20]. For example, Sorensen and Teske [14] investigated the active archaeal community in deep marine subsurface sediments from the Peru Margin and observed changes in activity and community composition over short distances in geochemically distinct zones. In another study on prokaryotic communities in deep-sea sediments collected at latitudes of 34°N to 79°N, archaeal abundances significantly increased from middle to high latitudes [13].

Despite the increasing number of studies, our knowledge of archaeal communities in deep-sea sediments of the central Pacific Ocean is still very limited. In this study, we assessed entire and potentially active archaeal communities at the seafloor of the Pacific Ocean. Sediments were sampled in the central Pacific during RV Sonne expedition SO248 along the 180° meridian. Sediment samples (0-1 cm below seafloor, cmbsf) were collected at nine sites from 27°S to 59°N exhibiting different depths of 3,258 to 5,909 meters below sea level (mbsl).

Entire and potentially active archaeal communities were assessed by Illumina tag sequencing of 16S rRNA amplicons generated from 16S rRNA genes and transcripts amplified by PCR and RT-PCR, respectively. In addition, functional profiles (artificial metagenomes) were predicted using Tax4Fun2 [21] to investigate functional changes of archaeal communities. The transpacific survey is of fundamental interest as it offers the opportunity to assess archaeal diversity, community composition, and its function in deep-sea sediments exhibiting a wide range of environmental conditions.

## 2. Material and Methods

### 2.1. Origin and Sampling of Sediments

**Figure 1:**
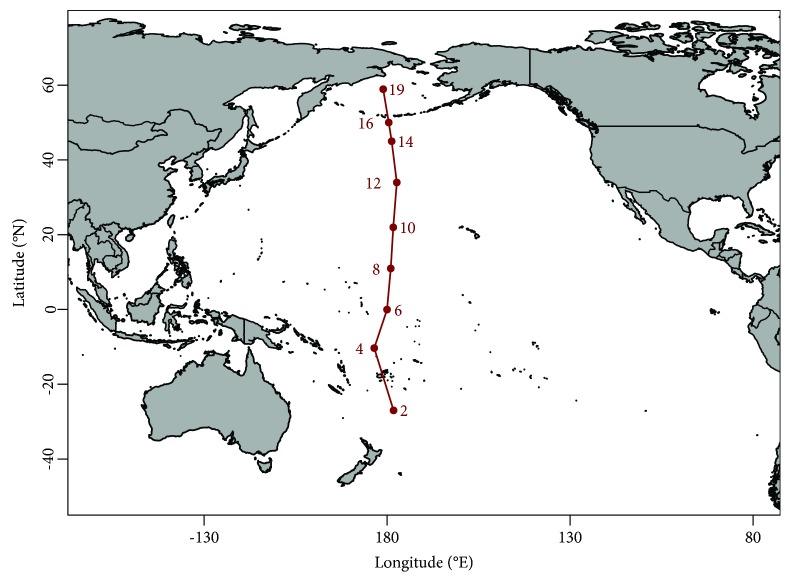
Map of the sampling sites in the Pacific Ocean. Samples were taken along the 180° meridian from 27°S to 59°N. For further information on sampling site characteristics, please see Supplementary Table S1.

Sediment samples were collected as described previously [22] in May 2016 during RV Sonne expedition SO248 along a transect ranging from Auckland, New Zealand, to Dutch Harbor, Alaska, USA (Figure 1, Supplementary Table S1). Briefly, sediment cores were taken using a multicorer (Octopus, Germany) at nine sites, exhibiting water depths between 3,258 and 5,909 mbsl. Sediment samples were collected using sterile, cutoff syringes and rhizons for porewater collection (Rhizosphere, Netherlands; [23]). Samples were immediately frozen and stored at -80°C for subsequent molecular analysis.

### 2.2. Measurement of Chlorophyll Concentrations and Geochemical Analyses of Bulk Sediments and Pore Waters

Chlorophyll concentrations were measured using a CTD-rosette equipped with a fluorometer (FluoroWetlabECO_AFL_FL, SN: FLNTURTD-4111). Data were recorded and stored using the standard software Seasave V 7.23.2 and processed using ManageCTD. The sum of chlorophyll concentrations measured in the top 500 m of the water column was used as a measure for the primary productivity in the water column at the different sampling sites. Raw data are available at PANGAEA: https://doi.pangaea.de/10.1594/PANGAEA.864673 [24].

The geochemical analysis of pore water and bulk sediments for all sites was described previously [22]. In brief, sediments were freeze-dried (Beta 1-8 LDplus, Christ, Germany) and homogenized using a Mixer Mill MM 400 (3 Hz, 50 min; Retsch, Germany). Total carbon (TC) and sulfur (S) content were measured with an elemental analyzer (Eltra CS-800, Germany) with a precision and accuracy of <3% (1*σ*). Inorganic carbon (IC) was analyzed using an acidification module (Eltra CS-580). The content of total organic carbon (TOC) was calculated by difference (TC–IC). Major and trace element analysis in sediments were performed by wavelength-dispersive X-ray fluorescence (XRF; Panalytical AXIOS plus). Nutrient concentrations of ammonium (NH_4_), nitrate (NO_3_), phosphate (PO_4_), and silicic acid in porewaters were determined photometrically using the Multiskan GO Microplate Spectrophotometer (Thermo Fisher Scientific, USA). The method for measuring ammonium was modified after Benesch and Mangelsdorf [25]. Nitrate was quantified with the method described by Schnetger and Lehners [26]. Concentrations of phosphate and silicic acid were determined following the protocol of Grasshoff et al. [27]. All measured environmental properties are provided in Supplementary Table S1.

### 2.3. Nucleic Acid Extraction and Sequencing

DNA extraction from sediment samples was performed using the DNeasy PowerSoil Kit (Qiagen, Germany) according to the manufacturer's instructions. RNA was extracted using the AllPrep DNA/RNA Mini Kit (Qiagen) following the manufacturer's protocol with some modifications as described previously [22]. The concentration and purity of DNA and RNA extracts were determined spectrophotometrically (NanoDrop 2000c; Thermo Fisher Scientific, USA).

Entire and potentially active archaeal communities were assessed by Illumina tag sequencing of 16S rRNA amplicons generated from 16S rRNA genes and transcripts amplified by PCR and RT-PCR, respectively. For RNA analysis, residual DNA was removed from extracted RNA by Turbo DNAse treatment, and the absence of DNA was confirmed by PCR using universal primers as described by Schneider et al. [28]. Subsequently, cDNA was generated from DNA-free RNA using the SuperScript III reverse transcriptase (Thermo Fisher Scientific) according to Wemheuer et al. [29] using the reverse primer without MiSeq adapter (5′-CCC GCC AAT TYC TTT AAG-3′; this study). Extracted DNA was treated with RNAse A as described by Schneider et al. [28] and purified using the GeneRead Size Selection kit (Qiagen) according to the manufacturer's instructions.

Archaeal 16S rRNA genes were amplified using the forward primer Arch514Fa (5′-GGT GBC AGC CGC CGC GGT AA-3′; [30]) and the reverse primer (5′-CCC GCC AAT TYC TTT AAG-3′; this study) with Illumina Nextera adapters attached for sequencing. Forward and reverse primers used for amplification were generated as follows: the Shannon index of diversity was calculated in a sliding window of 20 bp along all archaeal sequences in the SILVA database (SILVA 128 SSU Ref NR 99; [31]) to identify regions with low variability. The primer sequences were deduced from highly conserved regions and tested in silico using TestPrime (https://www.arb-silva.de/search/testprime/) and the SILVA 132 SSU Ref NR 99 [31] as reference database. The primer pair showed a 93.3% coverage of all archaea combined with a 99.9% specificity with 1 mismatch. It should be noted that the forward primer has been described previously [32] and that the primer pair will additionally amplify bacterial 16S rRNA gene sequences especially in the active fraction despite its high specificity for archaea. The PCR reaction (50 *μ*l) contained 10 *μ*l of fivefold Phusion GC buffer, 200 *μ*M of each of the four deoxynucleoside triphosphates, 1.5 mM MgCl_2_, 0.4 *μ*M of each primer, 2.5 *μ*l DMSO, 1 U of Phusion high-fidelity hot start DNA polymerase (Thermo Fisher Scientific), and approximately 25 ng of cDNA as template. The following thermal cycling scheme was used for amplification: initial denaturation at 98°C for 10 s and 10 cycles of annealing at 63°C for 30 s decremented by 1°C in each cycle, followed by extension at 72°C for 45 s, 20 cycles of denaturation at 98°C for 10 s, and annealing at 53°C for 30 s, followed by extension at 72°C for 15 s. The final extension was carried out at 72°C for 2 min. Each sample was subjected to three independent PCR reactions. Obtained PCR products were quantified by gel electrophoresis with the GeneRuler™ 1 kb DNA Ladder (Thermo Fisher Scientific) as standard, pooled in equal amount, and purified using the NucleoMag NGS Clean-up and Size Select kit (Macherey-Nagel, Germany). Obtained PCR products were quantified with a NanoDrop ND-1000 spectrophotometer (Thermo Fisher Scientific) and barcoded using the Nextera XT-Index kit (Illumina, USA) and the KAPA HiFi HotStart polymerase (Kapa Biosystems, USA). Sequencing was performed at the Göttingen Genomics Laboratory on an Illumina MiSeq system using the MiSeq reagent kit v3 (2 × 300 bp; Illumina).

### 2.4. Processing and Analysis of Illumina Datasets

Generated datasets were processed as follows: Trimmomatic version 0.36 [33] was initially used to truncate low-quality reads if quality dropped below 13 in a sliding window of 4 bp. Datasets were subsequently processed with USEARCH version 10.240 [34]. In brief, paired-end reads were merged and quality-filtered. Filtering included the removal of low-quality reads (maximum number of expected errors > 1 and more than 1 ambiguous base) and those shorter than 350 bp or longer than 450 bp. Processed sequences of all samples were concatenated into one file, dereplicated, and obtained unique sequences were denoised and clustered into zero-radius operational taxonomic units (zOTUs) with the *unoise3* algorithm. A de novo chimera removal was included in the unoise step. Additionally, the *unoise3* algorithm removed all unique sequences which appeared less than 8 times in the entire data set. Afterwards, remaining chimeric sequences were removed using the *uchime2* algorithm in high confidence mode with the most recent Silva database (SILVA SSU Ref 132 NR; [31]) as reference dataset. Afterwards, zOTUs were taxonomically classified by alignment employing BLASTn against the SILVA database [31] with an e value cutoff of 1*E* − 20. All nonarchaeal zOTUs were removed based on their taxonomic classification in the Silva database. Subsequently, processed sequences were mapped onto zOTU sequences to calculate the distribution and abundance of each zOTU in every sample using the *otutab* command with *maxrejects* and *maxaccepts* options disabled. Functional profiles were predicted using Tax4Fun2 [21] in reference mode (“Ref100NR”) with copy number correction enabled. To generate a phylogenetic tree, zOTUs were aligned with muscle (version 3.8.31 [35]), and the tree was calculated employing RaxML (version 8.2.10 [36]) using the GTRGAMMA model and a random seed of 1234. Sequence numbers during the main steps of processing are provided for each sample in Supplementary Table S2. The curated OTU table and the predicted functional profiles are provided as Tables S3 and S4, respectively.

### 2.5. Statistical Data Analysis

Statistical analyses were performed in R version 3.5.1 [37]. Differences were considered statistically significant with *p* ≤ 0.05. Entire and active archaeal communities were analyzed separately because different kits were used for nucleic acid extraction. Concentrations of ammonia and phosphate were below quantification limit at most sites of the transect and were not considered in the statistical analysis. Correlations between functional predictions and environmental conditions were tested by Spearman rank correlation using the *cor.test* function. Four samples in the RNA dataset (stations 2, 4, 10, and 12) were removed prior to statistical analysis due to low sequence numbers.

Alpha diversity indices (richness, Shannon index of diversity (SD), Faith's phylogenetic diversity (PD), and Michaelis-Menten Fit) were calculated using the R [37] packages *vegan* 2.4.-4 [38], *picante* version 1.7 [39], and *drc* version 3.0-1 [40]. OTU tables were rarefied to 9,058 (DNA dataset) or 4,941 (RNA dataset) sequences per sample prior alpha diversity analysis using the *rrarefy* function in *vegan* [38]. Shannon diversity was calculated using the *diversity* function in *vegan* [38]. Richness and Faith's PD were calculated using the *pd* function in *picante* [39]. Sample coverage was estimated using the Michaelis-Menten Fit calculated in R [37]. For this purpose, richness and rarefaction curves were calculated using the *rarecurve* and *specaccum* functions of the *vegan* package [38], respectively. The Michaelis-Menten Fit was subsequently calculated from generated rarefaction curves using the *MM2* model within the *drc* package [40]. All alpha diversity indices were calculated 100 times. The OTU table was rarefied in each iteration. The average of all iterations was used for further statistical analysis. Archaeal richness, diversity, and sample-wise coverage are provided in Supplementary Table S1.

Potential differences in community composition and function were investigated by permutational multivariate analysis of variance (PERMANOVA) using the *adonis* function within the *vegan* package [38]. Four different distance measures, i.e., unweighted and weighted Bray-Curtis (*binary* option in the *vegdist* function set to false and true, respectively) as well as unweighted and weighted UniFrac, were used. UniFrac values were calculated in R using the GUniFrac package [41]. In weighted approaches, low abundant members are barely considered, while unweighted dissimilarity measures provide insights into changes of the rare community. Additionally, incorporating taxonomy (UniFrac) provides insights into phylogenetic changes.

To limit the effect of the normalizing procedure, OTU tables were rarefied 999 times and the overall *p* value was calculated based on pseudo *F* statistics (https://github.com/vegandevs/vegan/issues/120) with epsilon correction (https://github.com/vegandevs/vegan/commit/8afee75a863473c49b5b7468c3cb9acf3c9e28c9). The R code is provided as Supplementary Data S1. For functional differences, Bray-Curtis distances were calculated between the samples using the *vegdist* function of the *vegan* package [38].

## 3. Results and Discussion

### 3.1. Geochemical Characteristics of the Sediment Samples

The characteristics of the sampling sites were described in detail by Pohlner et al. [22]. Generally, most parameters showed an increasing trend toward the north with highest concentrations in the Pacific subarctic region and the Bering Sea. Chlorophyll concentrations (summed over the upper 500 m of the water column) ranged from 41.9 mg/m^3^ to 122.9 mg/m^3^, with higher concentrations at the northernmost sites. This indicates a higher primary production in the water column at these stations. The lowest ferric oxide and manganese dioxide concentrations were measured at stations 10 and 12, respectively, whereas the highest concentrations were determined at station 2. The TOC content as a general indicator for nutrient availability fluctuated around 0.6% of the sediment dry weight. The highest TOC content of 1.3% was found in the Bering Sea at 59°N. The TOC values observed here are comparable to those of previous studies on marine sediments [11, 42, 43].

**Figure 2:**
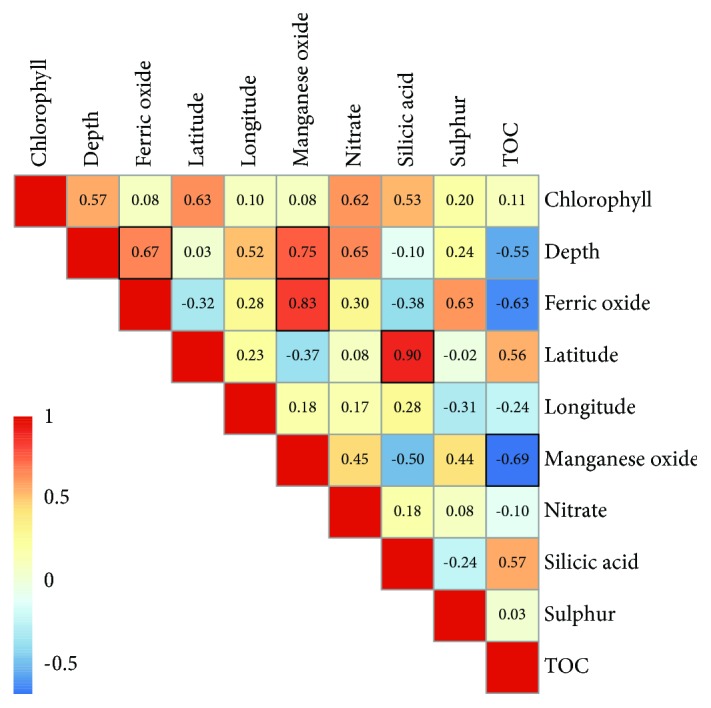
Correlations between environmental properties. The Spearman's rho is provided in each box. Significant correlations (*p* ≤ 0.05) are highlighted in black.

Consistent with previous work [44–46], several environmental properties were significantly correlated with each other (Figure 2). For example, ferric oxide concentration significantly increased with water depth. Moreover, ferric oxide and manganese dioxide concentrations as well as TOC content and manganese dioxide were significantly correlated. In contrast to our observation, no significant correlations between TOC and manganese or iron were detected in a study on shallow marine sediments from Jinzhou Bay, China [47]. In another study on anaerobic mangrove sediments, positive correlations among TOC and iron and negative correlations of manganese with TOC were observed [48]. Montalvo et al. [46] found a high relationship between iron and manganese in sediments of the deltaic lagoon-river system of the Palizada River (Mexico), which is supported by our results.

### 3.2. Archaeal Alpha Diversity Is Affected by Few Environmental Factors

**Figure 3:**
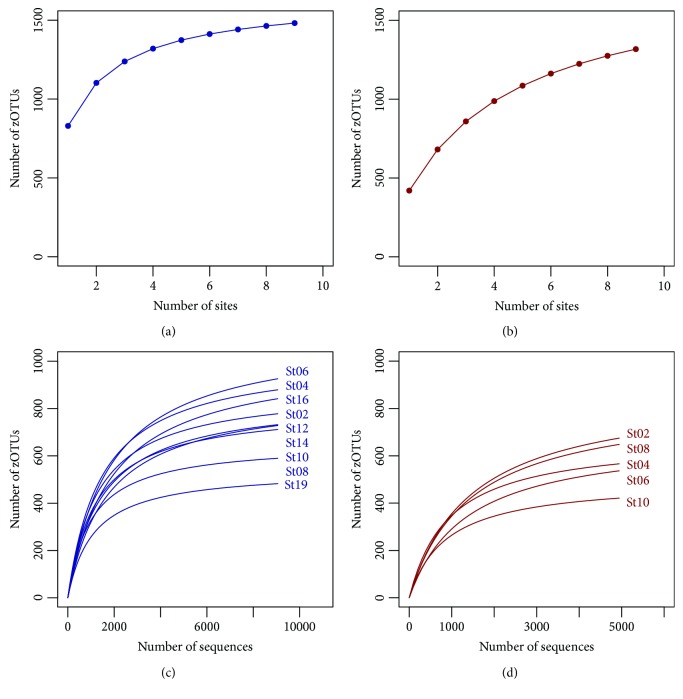
Species accumulation and rarefaction curves calculated for the entire (a, c) and potentially active (b, d) archaeal community. Entire and active archaeal communities are colored in blue and red, respectively.

Entire and potentially active archaeal communities in deep-sea sediment samples were assessed by Illumina (MiSeq) sequencing targeting archaeal 16S rRNA genes and transcripts, respectively. After removal of low-quality reads, PCR artefacts (chimeras), and contaminations, a total of 164,523 high-quality reads were obtained. Sequence numbers per sample varied between 9,058 (station 2) and 16,061 (station 19) for the entire community and 50 (station 2) and 18,069 (station 16) for the active community, respectively (Supplementary Table S3). Four RNA samples (stations 2, 4, 10, and 12) were not included in the statistical analysis due to low sequence numbers. Obtained sequences were grouped into 1,469 zOTUs. Species accumulation curves indicated that 89% and 72% of all zOTUs were recovered from the entire and active archaeal community (Figures 3(a) and 3(b)). Rarefaction analysis and sample-wise coverage based on Michaelis-Menten Fit confirmed that the majority of the entire and active archaeal communities (88.1% and 81.8%, respectively) were recovered by the surveying effort (Figures 3(c) and 3(d), Supplementary Table S1).

In order to study changes in archaeal richness and diversity along the investigated transect, we calculated richness, diversity, and phylogenetic diversity. At DNA level, archaeal richness and diversity ranged from 497 to 939 and from 5.1 to 6.1, respectively (Supplementary Table S1). At RNA level, archaeal richness and diversity varied between 432 and 684 and between 5.1 and 5.6, respectively. The phylogenetic diversity ranged from 389.1 to 523.9 (DNA level) and from 401.9 to 525.2 (RNA level). Recently, Zhang et al. [18] investigated archaeal communities from two deep-sea sediments of inactive hydrothermal vents in the Southwest India Ridge and observed differences in archaeal diversity between these samples. The authors concluded that environmental parameters have a significant impact on archaeal communities.

**Figure 4:**
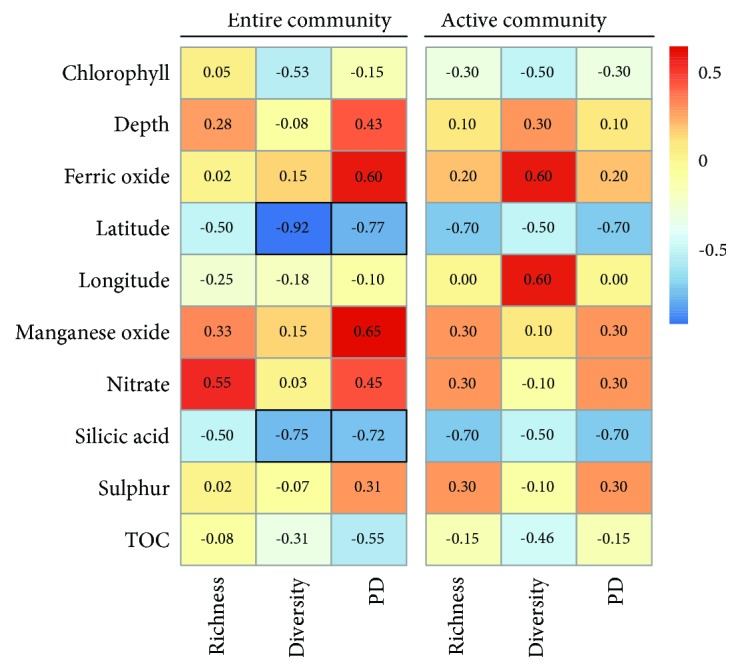
Correlations between environmental properties and alpha diversity measures. The Spearman's rho is provided in each box. Significant correlations (*p* ≤ 0.05) are highlighted in black.

Consequently, we tested for significant correlations between alpha diversity measures and environmental conditions (Figure 4). The diversity and the phylogenetic diversity of the entire archaeal community were negatively correlated with latitude and silicic acid. These results are in line with a recent study on ammonia-oxidizing archaea in sediments of the eastern China marginal seas [49]. Here, the diversity of these archaea significantly decreased with latitude and silicic acid. In contrast to our study, water depth was the main driver of the archaeal phylogenetic diversity in surface sediments from the northern part of the South China Sea [50]. In another study on archaeal diversity in deep-sea sediments from 11 sites, archaeal richness was significantly affected by temperature and latitude, but not by water depth [51]. However, the authors of the first study and we analyzed the entire archaeal community, whereas Cao et al. [50] investigated only ammonia-oxidizing archaea, which might explain the contrasting results. In contrast to our results observed for the entire community, we did not find any significant correlations for alpha diversity measures of the active archaeal community, which might be related to the low sample number.

### 3.3. Archaeal Taxa Exhibit a Distinct Biogeographical Distribution

**Figure 5:**
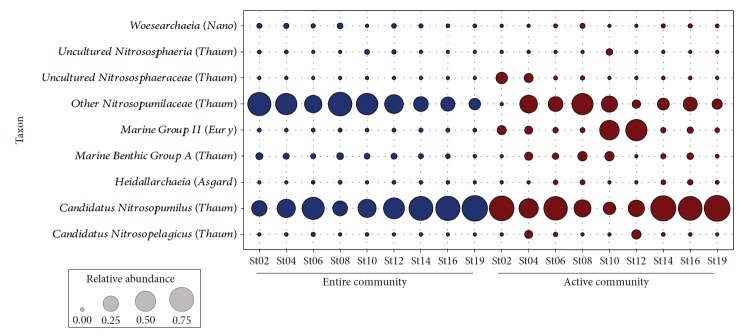
Community composition of entire and active archaeal communities along the investigated transect. Entire and active archaeal communities are colored in blue and red, respectively.

*Thaumarchaeota* accounted for 99.63% of the entire archaeal community (Figure 5). Other minor abundant community members were related to *Nanoarchaeaeota* (0.31%), *Euryarchaeota* (0.05%), and *Asgardaeota* (0.01%). The potentially active archaeal community was dominated by the *Thaumarchaeota* (86.48%), but higher abundances of *Euryarchaeota* (13.23%) were detected compared to the entire archaeal community. Other rare archaeal phyla in the active community were *Asgardaeota* (0.20%) and *Nanoarchaeaeota* (0.09%). The dominance of the two phyla *Thaumarchaeota* and *Euryarchaeota* is consistent with previous studies on archaeal communities in marine deep-sea sediments, although divergences of the relative abundances of these phyla were detected with respect to sampling sites [11, 13, 18].

At order level, members of the *Nitrosopumilales* accounted for 98.59% (DNA level) and 81.91% (RNA level) of all sequences observed in this study. Within this order, archaea belonging to the *Nitrosopumilaceae* (DNA level 43.98%; RNA level 21.63%) and Candidatus *Nitrosopumilus* (DNA level 54.59%; RNA level 59.11%) were predominant. High relative abundances of *Nitrosopumilus* were also observed in a recent study on bacterial and archaeal communities in the Mediterranean Sea [52]. Here, between 62% and 76% of the archaeal sequences were affiliated to *Nitrosopumilus*. In a recent study on ammonia-oxidizing archaea and bacteria, the *Nitrosopumilus* lineage was predominant in the archaeal community, but the abundance of this lineage varied significantly between the sites investigated [49]. In contrast, lower abundances of *Nitrosopumilus* were observed in two studies on archaeal communities in pan-Arctic Ocean sediments [17] and in deep-sea sediments of inactive hydrothermal vents in the Southwest India Ridge [18]. The high abundance of ammonia-oxidizing archaea (i.e., *Nitrosopumilus*) in our study might serve as an indicator for rather oligotrophic conditions of the investigated sediment samples.

Interestingly, archaea of the order *Nitrososphaerales* were only observed in the active community (Figure 5, Supplementary Table S3). In addition, members of the Marine Group II were found in higher abundances in the active (13.23%) compared to the entire community (0.05%). Previous studies showed that archaea of the Marine Group II were widely distributed in global oceans [53–55], but their abundances can vary greatly over time [56]. Recently, Liu et al. [55] observed the dominance of Marine Group II in planktonic archaea throughout the water column of the South China Sea. In contrast, Marine Group II members were almost absent in a study on archaeal communities in the German Bight [9]. Archaea of the Marine Group II have unique patterns of organic carbon degradation (as reviewed by Zhang et al. [57]). However, our understanding of ecological and biogeochemical functions of these archaea is still very limited, as there is no pure culture of Marine Group II available [55–57].

The abundances of the archaeal groups differed between the stations. For example, higher abundances of Candidatus *Nitrosopumilus* in the entire archaeal community were recorded at stations 6, 14, 16, and 19. The Marine Benthic Group A had higher abundances at stations 2 and 8 but was almost missing at station 19, whereas higher abundances of the *Nitrosopumilaceae* were observed at stations 2, 4, 8, and 10. These results might be related to observed differences in environmental conditions and/or the overlying water column, as these factors can influence the distribution and community structure of archaeal communities in marine sediments [11, 13, 20]. For example, Danovaro et al. [13] showed that the abundances of the two dominant groups MG-I *Thaumarchaeota* and MG-II *Euryarchaeota* increased from middle to high latitudes. This was more pronounced for the MG-I *Thaumarchaeota*. They further observed that the two groups displayed a different sensitivity toward changes in the main environmental drivers. The authors concluded that this might favour their coexistence by reducing niche overlap and competition for available resources, which might also play a role in our study.

### 3.4. Primary Production in the Water Column Is the Main Driver of Sediment Archaeal Community Composition

**Figure 6:**
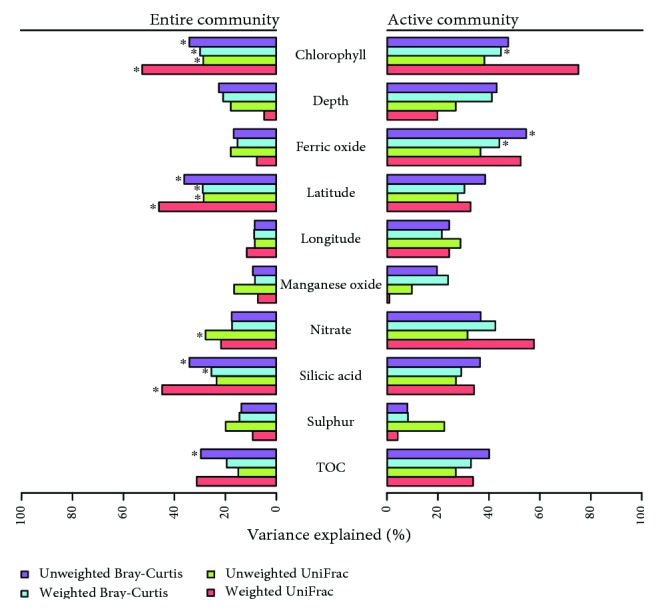
Results of the beta-diversity analysis between entire as well as active archaeal community composition and environmental properties. Correlations between community composition and each environmental property were tested by PERMANOVA using four different distance measures. Significant correlation (*p* ≤ 0.05) are marked with a star.

The composition of the active archaeal community was only affected by ferric oxide and chlorophyll content and only when using weighted and/or unweighted Bray-Curtis dissimilarities (Figure 6). We detected no effect of manganese oxide on both the active and the entire archaeal community composition. These results are only partly in line with the findings of Jorgensen et al. [11] who showed that the content of iron and manganese within arctic sediments was strongly related to microbial community structure. In contrast, Algora et al. [58] observed that manganese but not ferric oxide concentrations were significantly correlated with structural shifts of the archaeal community in marine sediments. Recently, Wang et al. [59] investigated the diversity and spatial distribution of prokaryotic communities in surface sediments of the Arctic Ocean and showed that archaeal community composition was mainly determined by metallic ions. According to Durbin and Teske [42], organic-lean marine sediments in deep marine basins and oligotrophic open ocean locations are inhabited by distinct lineages of archaea. The authors suggested that different combinations of electron donor and acceptor concentrations along the organic-rich/organic-lean spectrum result in distinct archaeal communities.

In the present study, several measured environmental properties such as latitude or chlorophyll content as proxy for primary production affected the composition of the entire archaeal community. This is partly in accordance with previous studies on sediment archaea [49, 50, 59, 60]. For example, Wang et al. [59] showed that the archaeal community composition in surface sediments of the Arctic Ocean was influenced by latitude, water depth, and TN. In a previous study on archaeal communities in sediments of the North Chinese Marginal Seas, these communities were significantly affected by temperature, dissolved oxygen of bottom water, and chlorophyll *a* in sediments [60]. A similar significant impact of dissolved oxygen of bottom water on ammonia-oxidizing archaeal community composition was observed by Liu et al. [49]. In another study on ammonia-oxidizing archaeal communities in surface sediments of the Western Pacific, these communities were significantly affected by ammonium, water depth, and nitrite [50]. We postulate that the contrasting results in our and the abovementioned studies are related to differences in sampling sites and sampling time, as these factors can affect sediment archaeal communities [49, 50, 56]. Another possible explanation is that most previous studies have focused on ammonia-oxidizing archaea only [12, 49, 50], whereas we assessed the entire archaeal community.

We further recorded differences between the four dissimilarity indices used for beta-diversity analysis. TOC and nitrate affected the entire archaeal community composition, but only when applying unweighted Bray-Curtis or unweighted UniFrac dissimilarities, respectively. A significant influence of TOC on the ammonia-oxidizing archaeal community composition was also observed in a study on ammonia-oxidizing archaea and bacteria in sediments of the Yellow River estuary [61]. Partly in line with our study, water depth, ammonium, and nitrate concentration affected the composition of ammonia-oxidizing archaeal communities in deep-sea sediments of the Eastern Indian Ocean [12]. Overall, the results of the above mentioned research and our study indicate that multiple environmental properties are important drivers of archaeal communities. However, our observations highlight the importance of using different dissimilarity indices to gain better insights into environmental drivers of archaeal communities in deep-sea sediments. As more significant correlations were observed when weighted UniFrac dissimilarities were used, we suggest that the taxonomic relationship of the zOTUs is a important driver of community structuring.

### 3.5. Archaeal Community Function Is Strongly Correlated to Primary Production

**Figure 7:**
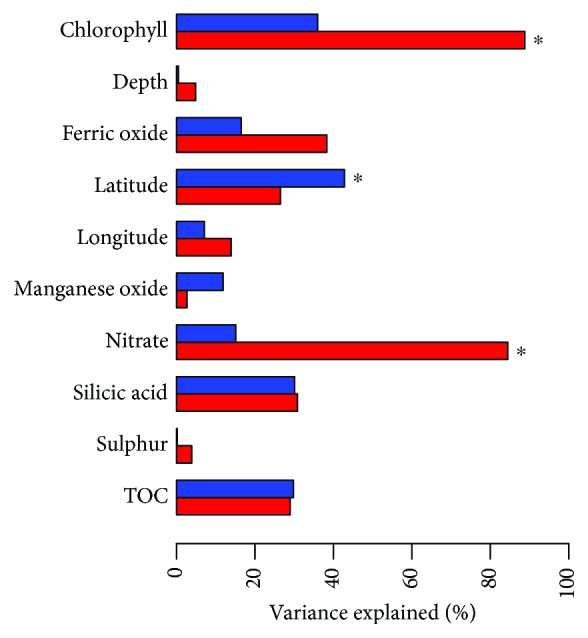
Correlations between functional profiles predicted from entire and active archaeal community composition and environmental properties. Correlations between community composition and each environmental property were tested by PERMANOVA. Significant correlation (*p* ≤ 0.05) are marked with a star.

To investigate potential changes in community function along the investigated transect, functional profiles for the archaeal community were predicted from 16S rRNA data using Tax4Fun2 [21]. Approximately 38% of all zOTUs, representing 45% of all the sequences obtained, were used in the prediction. This indicates that a large proportion of the archaeal community in Pacific deep-sea sediments is still completely unknown. We found a significant correlation between latitude and the functional profile predicted for the entire community (Figure 7). In contrast, nitrate and chlorophyll contents were significantly correlated to function of the potentially active community. Primary production is believed to play an important role in controlling the metabolism and standing stocks of benthic assemblages in the deep sea due to the magnitude of organic carbon export from the surface waters to the deep-sea floor through sinking particles [13]. We further hypothesize that our observations can be explained by the high abundance of Candidatus *Nitrosopumilus* in the archaeal community. A previous study showed that *N. maritimus* takes part in both nitrogen and phosphorus metabolism [62]. Two other studies on *N. maritimus* revealed a wide range of cellular, genomic, and physiological features that reflect an oligophilic lifestyle [63, 64]. The results of these studies further suggest that *N. maritimus* play an important role in the biogeochemical cycles of nitrogen and carbon.

### 3.6. Study Limitations and Future Studies

Several technical and biological limitations of the present study must be considered. First, the number of samples is relatively low compared to other studies (e.g., [11, 13]; but see [12, 15, 50, 59, 60]). However, higher sample numbers are usually the result of several independent sampling campaigns, whereas all samples investigated in the present study were taken during one cruise within one month. Secondly, RNA is less stable than DNA and, hence, its rapid degradation might have affected the results of this study. The sampling procedure used in this study maintains the structure of the sediment during collection and thus minimizes any perturbation. In addition, sediment samples were immediately flash-frozen after sampling and kept frozen at -80°C or on dry ice until RNA extraction.

Another caveat is the low sequencing depth for the active archaeal community. An enhanced sequence coverage would permit more in-depth and solid analyses. Nonetheless, the low depth observed for the active community is most likely caused by a low contribution of Archaea to the active prokaryotic community as the four samples displaying low sequence numbers contained less than 2% archaeal and more than 98% bacterial sequences. In addition, this study highlights the importance of examining both entire and potentially active archaeal communities, as the environmental properties affected the composition of the entire and the active archaeal community in a different manner. To date, most previous studies have focused on assessing the archaeal community at DNA level [51, 59, 60] and/or ammonia-oxidizing archaea only [12, 49, 50]. Consequently, future studies should investigate both entire and active archaeal communities to obtain a more holistic picture of archaeal communities in deep-sea sediments.

Finally, it must be noted that functional predictions cannot replace metagenomic or metatranscriptomic sequencing. The high number of OTUs unused in the Tax4Fun2 prediction, likely caused by missing reference genomes, highlights the importance of these approaches. However, the results of our study can serve as template for further metagenomic or metatranscriptomic studies on archaeal communities in deep-sea sediments.

## 4. Conclusion

In the present study, we assessed both entire and active archaeal communities along the 180° meridian of the central Pacific Ocean. The archaeal taxa differed in their relative abundances between the sites. We further observed that the composition of the archaeal community was strongly correlated to the primary production in the water column. Only latitude was significantly correlated with functional profiles predicted at the entire community level, whereas primary production and nitrate content were significantly correlated to function of the potentially active community. Community composition and predicted functional profiles were driven by different environmental conditions. This finding indicates that changes in archaeal community composition are not necessarily linked to changes in overall community function. Our results further suggest that benthic archaea in the Pacific Ocean are important contributors to the carbon and nitrogen cycle. Nonetheless, further studies are needed to address their ecological role and ecosystem function in the investigated sediments.

## Data Availability

Sequence data were deposited in the sequence read archive (SRA) of the National Center for Biotechnology Information (NCBI) under the accession numbers SRP151826 (BioProject PRJNA479333). Individual accession numbers for each sample are provided in Supplementary Table S2.

## Supplementary Materials

Supplementary 1 Data S1: R code example for PERMANOVA analysis with rarefied OTU tables.

Supplementary 2 Table S1: sampling site characteristics and alpha diversity values.

Supplementary 3 Table S2: sequence characteristics and accession numbers.

Supplementary 4 Table S3: OTU table.

Supplementary 5 Table S4: functional predictions made with Tax4Fun2.
